# RORα Regulates Cholesterol Metabolism of CD8^+^ T Cells for Anticancer Immunity

**DOI:** 10.3390/cancers12071733

**Published:** 2020-06-29

**Authors:** In Kyu Lee, Hyerin Song, Hyerim Kim, Ik Soo Kim, Na Ly Tran, Sang-Heon Kim, Seung Ja Oh, Ji Min Lee

**Affiliations:** 1 Center for Biomaterials, Biomedical Research Institute, Korea Institute of Science and Technology (KIST), Seoul, 02792, Korea; inkyulee@kist.re.kr (I.K.L.); 618004@kist.re.kr (N.L.T.); skimbrc@kist.re.kr (S.H.K.); 2 Department of Molecular Bioscience, College of Biomedical Sciences, Kangwon National University, Chuncheon 24341, Korea; shyerin521@gmail.com; 3 Program in Nanoscience and Technology, Graduate School of Convergence Science and Technology, Seoul National University, Seoul 08826, Korea; hyerimkim@snu.ac.kr; 4 Department of Pathology, Massachusetts General Hospital and Harvard Medical School, Boston, MA 02114, USA; iskim@mgh.harvard.edu; 5 Division of Bio-Medical Science & Technology, Korea University of Science and Technology (UST), Daejeon 34113, Korea

**Keywords:** Cholesterol, CD8^+^ T cell, RORα, NF-κB

## Abstract

Retinoic acid-related orphan receptor α (RORα) functions as a transcription factor for various biological processes, including circadian rhythm, inflammation, cancer, and lipid metabolism. Here, we demonstrate that RORα is crucial for maintaining cholesterol homeostasis in CD8^+^ T cells by attenuating NF-κB transcriptional activity. Cholesterol sulfate, the established natural agonist of RORα, exhibits cellular cytotoxicity on, and increased effector responses in, CD8^+^ T cells. Transcript analysis reveals that the suppression of RORα leads to the upregulation of NF-κB target genes in T cells. Chromatin immunoprecipitation analysis was used to determine the corecruitment of RORα and histone deacetylase (HDAC) on NF-κB target promoters and the subsequent dismissal of coactivators for transcriptional repression. We demonstrate that RORα/HDAC-mediated attenuation of NF-κB signaling controls the balance of cholesterol metabolism in CD8^+^ T cells, and that therapeutic strategies targeting this epigenetic regulation could be beneficial to the treatment of solid tumors including colon cancers.

## 1. Introduction

RORα, a member of the orphan nuclear receptor (ONR) family, plays an essential role in various physiological functions such as cellular differentiation, metabolic pathway, inflammation, and cancer [1,2,3,4,5,6,7]. A recent study suggested that targeting the RORα downstream signaling of cholesterol metabolism modulates osteoarthritis pathogenesis [8], which indicates that the regulation of intracellular cholesterol levels is crucial for the pathogenesis of inflammation. We previously reported that RORα has a tumor-suppressive function by transrepressing Wnt/β-catenin signaling, leading to the suppression of colorectal cancer growth, and by increasing p53 protein stability, leading to apoptosis of cancer cells in response to DNA damage [9,10,11]. RORα also upregulates the expression of many target genes for the regulation of circadian rhythm, whereas other ONRs, REV-ERBs, reduce the circadian targets [12]. Further, RORα regulates the expression of Patterns recognition receptors (PRRs) upon the circadian rhythm [13]. Although the various functions of RORα as a negative regulator of the NF-κB signaling pathway have been shown [3,14], the molecular mechanisms associated with antitumor immunity remain to be determined.

The pharmacological activation of RORα by a synthetic agonist SR1078 and cholesterol sulfate (CS) activates RORα driven transcription, while the RORα-specific inhibitor SR3335 functions as a significant selective inverse agonist [15,16]. These compounds were initially identified based on their abilities to enhance or inhibit the constitutive activity of RORα in a GAL4-RORα ligand-binding domain (LBD) cotransfection assay. It was clear that SR1078, CS, and SR3335 dose-dependently compete with binding to the RORα LBD. Although SR1078 behaves as a dual agonist of RORα and RORγ, it has been suggested that CS is a natural selective ligand for RORα [17]. In addition, the RORα selectivity of SR3335 confirmed that SR3335 exhibits no activity on RORβ, RORγ, Liver X Receptor (LXR) α, FXR, or any other receptors. The critical role has emerged of ONRs and nuclear receptors (NRs), including RORα, in regulating metabolic gene networks with therapeutic potential for the treatment of metabolic syndrome [18]. Targeting the ONR or NR substrate directly via their LBD, as opposed to the modifying enzymes affecting receptor oligomeric state or DNA binding affinities, may prove to be a preferable strategy with reduced off-target effects. Thus, we utilized a selective agonist and inverse agonist of RORα as chemical tools to probe the function of RORα in the crosstalk between cholesterol metabolism and anticancer effects.

Many clinical data demonstrate that increased T cell infiltration in tumors improves cancer patient prognoses [19,20,21]. CD8^+^ T cells called cytotoxic T cells have been shown to play critical roles in antitumor immunity [22]. Activated CD8^+^ T cells induce cancer apoptosis by either secreting cytotoxic molecules or by direct cell-to-cell contact [23]. However, CD8^+^ T cells often lose their effector function, thereby limiting antitumor immunity in a tumor microenvironment. Therefore, there are unmet clinical needs that reactivate the antitumor potency of CD8^+^ T cells in cancer treatment [24]. Various methods have been applied to revitalize the effector functions of CD8^+^ T cells in a tumor microenvironment [25]. Previous reports have demonstrated that the function of T cells can be regulated by the lipid concentration in the plasma membrane [26,27]. As one of the major components of membrane lipids, cholesterol contributes to the clustering of T cell receptors (TCRs) and the formation of immunological synapses [28]. Therefore, potentiating the antitumor response of CD8^+^ T cells by modulating cholesterol metabolism has been attempted. A recent study showed that the inhibition of Acetyl-CoA acetyltransferase 1 (Acat1), a cholesterol esterification enzyme, enhances the effector function of CD8^+^ T cells [29]. Even though the studies proposed modulating cholesterol as a potential strategy for cancer immunotherapy, the underlying molecular mechanism related to the regulation of lipid synthesis is poorly understood.

Here, we report that RORα functions as an epigenetic regulator to maintain the balance of the cholesterol metabolism in CD8^+^ T cells using human primary T cell with a specific agonist or antagonist of RORα [16,30,31]. Natural and artificial agonists of RORα exhibit CD8^+^ T cell effector responses, while the repression of RORα in T cells remarkably decreases T cell activation. ChIP analysis reveals that RORα acts as a transcriptional repressor of cholesterol esterification and cholesterol efflux genes by attenuating NF-κB signaling in T cells, indicating that RORα plays a key role in maintaining cholesterol homeostasis to prevent cancer progression. Our data demonstrate that RORα is critical for preserving cholesterol metabolism via the attenuation of NF-κB signaling for the activation of CD8^+^ T cells.

## 2. Results

### 2.1. Enhanced RORα Activities Improves Cholesterol Metabolism in T Cells

**Figure 1:**
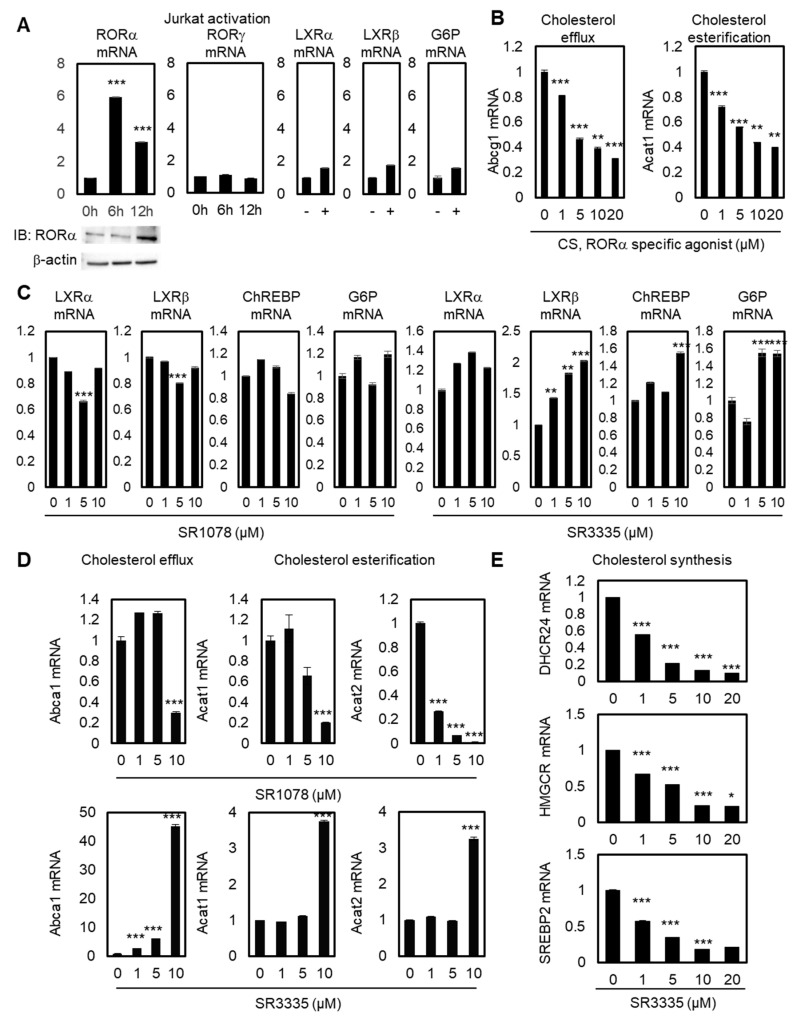
Increased RORα activity is crucial for maintaining cholesterol levels in T cells. (**A**) Transcriptional levels of *RORα*, *RORγ*, *LXRα*, *LXRβ*, and *G6P* in stimulated Jurkat cells by activation times (+: 24 hours). Protein levels of RORα were detected by immunoblot analysis in stimulated Jurkat cells. The *p*-value was calculated by a *t*-test (*** *p* < 0.001). (**B**) Transcriptional levels of *Abcg1* and *Acat1* mRNAs in Jurkat cells treated with CS. The *p*-value was calculated by a *t*-test (** *p* < 0.01, *** *p* < 0.001). (**C**) Transcriptional levels of *LXRα*, *LXRβ*, *ChREBP*, and *G6P* in Jurkat cells treated with SR1078 or SR3335. The *p*-value was calculated by a *t*-test (** *p* < 0.01, *** *p* < 0.001). (**D**) Transcriptional levels of *Abca1*, *Acat1*, and *Acat2* in Jurkat cells treated with SR1078 or SR3335. The *p*-value was calculated by a *t*-test (*** *p* < 0.001). (**E**) Transcriptional levels of cholesterol synthesis genes *DHCR24*, *HMGCR*, and *SREBP2* in Jurkat cells treated with CS. The *p*-value was calculated by a *t*-test (* *p* < 0.05, *** *p* < 0.001).

To explore the function of RORα in T cells by regulating cholesterol levels, we first checked the reprogramming of ROR family genes during Jurkat cell activation. RORγ is known as the master transcription factor of IL-17 expression and Th17 cells, while the function of RORα in T cell regulation is largely unknown [32,33]. Jurkat cells were activated with antibiotin MACSiBead particles and biotinylated antibodies against human CD2, CD3, and CD28. Upon activation of Jurkat cells, *RORα* mRNA and related protein levels were significantly upregulated after 6 and 12 hrs, whereas *RORγ* mRNA levels did not change (Figure 1A). To test the specificity of the upregulation of RORα, we checked the mRNA levels of *LXRα*, *LXRβ*, and their target genes, such as *Glucose 6-phosphate* (*G6P*). Their mRNA levels were not significantly increased by Jurkat cell activation; therefore, these data illustrate the possibility of selective activation of RORα by T cell activation. Modulating cholesterol metabolism has been proven to potentiate the antitumor response of CD8^+^ T cells, and ACAT1 has been shown to be the major enzyme of cholesterol esterification in CD8^+^ T cells [28]. To test the function of RORα in the cholesterol metabolism of T cells, we also checked the mRNA levels of cholesterol esterification and cholesterol efflux genes with stimulated RORα activities using a potent agonist, cholesterol sulfate (CS). Activating RORα led to decreased mRNA levels of *ATP-binding cassette transporters G1* (*Abcg1*) and *Acat1*, supporting the hypothesis that increased cholesterol levels by downregulation of cholesterol esterification and efflux genes in T cells can potentiate the effector function of T cells (Figure 1B). Despite playing a role in cholesterol efflux, ABCA1 and ABCG1 are the major LXRα downstream pathway of macrophages [34]; we assumed that the mRNA levels of *Abcg1* and *Acat1* would be affected mainly via the downstream pathway of RORα. First, in order to test whether LXRα, LXRβ, and their downstream pathway were regulated by modulators of RORα, qRT-PCR was performed with these genes after the treatment of agonist and antagonist of RORα in Jurkat cells. General targets of the lipid metabolism pathway controlled by LXRα, such as *carbohydrate-responsive element-binding protein* (*ChREBP*) and *G6P*, did not change under treatment with an agonist or antagonist of RORα, suggesting that cholesterol metabolism in T cells is principally regulated through RORα (Figure 1C). Under T cell activations, the activation of TCR stimulates cholesterol synthesis by affecting the transcription of essential enzymes in the cholesterol synthesis pathway. ACAT1 plays key regulatory roles in maintaining intracellular cholesterol stability by inducing cholesterol esterification in order to decrease the levels of available cholesterol in T cells. Cholesterol efflux genes such as ABCA1/G1 outflow cholesterol from T cells to high-density lipoprotein (HDL) and lipid poor apolipoprotein A1 (apoA1) molecules. Therefore, both cholesterol esterification and efflux genes may reduce accessible cholesterol levels in T cells, and the downregulation of these targets should lead to the upregulation of cholesterol levels in the plasma membrane of CD8^+^ T cells. Increased cholesterol levels eventually enhance TCR clustering and signaling, and lead to the efficient formation of immunological synapses between T cells and cancer cells. Decreased mRNA levels of target genes related to cholesterol efflux and esterification in T cells were additionally confirmed by treatment of SR1078, a dual agonist of RORα and RORγ (Figure 1D). The selective agonist for RORα has more specific enhancing effects on the downstream target genes of cholesterol metabolism in T cells. Furthermore, inhibiting RORα using specific antagonist small molecule SR3335 enhances mRNA levels of *Abca1*, *Acat1*, and *Acat2* (Figure 1D). Regarding the mRNA levels of *Abca1* and *Acat1*, only 10μM of SR1078 had a significant effect on target genes. By contrast, SR3335 significantly decreased gene sets of cholesterol biosynthesis, *DHCR24*, *HMGCR*, and *SREBP2* (Figure 1E), indicating markedly reduced cholesterol levels in T cells by affecting RORα activities. These data indicate that RORα triggers increased levels of cholesterol for T cell activation.

### 2.2. RORα Acts as a Corepressor for Cholesterol Esterification via NF-κB-Dependent Promoter Regulation

**Figure 2:**
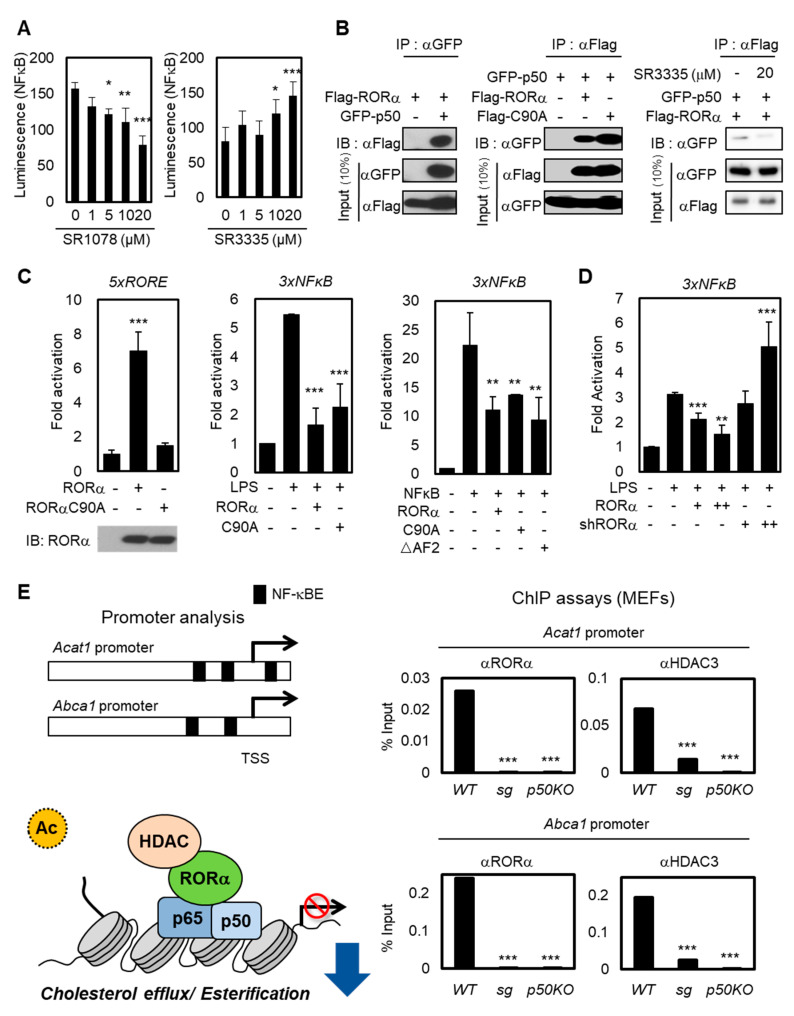
RORα potentiates T cell cholesterol synthesis via NF-κB inhibition. (**A**) NF-κB-luciferase reporter-expressed Jurkat cells were treated with SR1078 or SR3335 at doses of 1, 5, 10, and 20 μM for 1 day. Luciferase was reacted by the Luciferase Assay System and measured using a microplate reader. The *p*-value was calculated by a *t*-test (* *p* < 0.05, ** *p* < 0.01, *** *p* < 0.001). (**B**) Co-immunoprecipitation assays between RORα and p50, NF-κB component (left). The binding affinity of RORα WT or C90A with p50 was assessed in 293T cells (middle). The binding between RORα and p50 was assessed with SR3335 treatment in 293T cells (right) (**C**) The introduction of RORαC90A decreased the transcriptional activation of the 5XRORE luciferase reporter (left). Compared to RORE, the introduction of RORα WT, C90A, and ΔAF2 mutants had the same effect of transrepression on the 3XNFκB luciferase reporter (middle and right). The *p*-value was calculated by a *t*-test (** *p* < 0.01, *** *p* < 0.001). (**D**) Effects of RORα knockdown on 3XNFκB luciferase reporter in 293T cells treated with LPS (shRORα, +: 0.5μg/μL, ++: 2μg/μL). (**E**) The illustration represents the location of NF-κB elements on *Acat1* and *Abca1* promoter. Proposed model of RORα recruitment with HDAC3 and binding on the NF-κB target promoters (left). ChIP assays on *Acat1* or *Abca1* promoters in WT, ROR KO (*sg*), or *p50* KO MEFs (right). The *p*-value was calculated by a *t*-test (*** *p* < 0.001).

The NF-κB family consists of p65/Rel A, p50/NF-κB1, p52, Rel B, and c-Rel. Among them, the p50-p65 heterodimer is the most abundant [35,36]. The heterodimer p50-p65 serves as a master regulator to maintain inflammatory responses in immune cells, and the NF-κB signaling enhances specifically the expression of the human *Acat1* gene in differentiating monocytes [37]. To explore how the transcriptional activities of cholesterol esterification and efflux genes are changed by RORα activation or repression, we detected NF-κB luminescence in Jurkat cells with RORα agonist or antagonist (Figure 2A). While treatment using RORα agonist SR1078 substantially suppressed the luciferase activity, SR3335 enhanced the NF-κB luciferase activity. Then, we performed a co-immunoprecipitation analysis to examine the interaction between NF-κB protein and RORα WT or DNA binding domain (DBD) mutant C90A (Figure 2B,C). p50 is the predominant regulatory subunit of the NF-κB complex; it interacts directly with other components rather than p65 [38]. As shown in Figure 2B, roughly the same amounts (10% input) of each RORα and p50 in all conditions were loaded, but the interactions between RORα and p50 were detected only when both plasmids were coexpressed. Further, downregulating RORα activities with SR3335 treatment decreased the binding between RORα and p50 (Figure 2B). These data allowed us to exclude the possibility that RORα transrepresses NF-κB-mediated transcription in a DNA-binding-dependent manner. To evaluate the underlying molecular mechanism of RORα on NF-κB signaling, we performed a reporter assay using luciferase plasmid containing 3x NF-κB response element (RE) in the regulatory region. While overexpression RORα WT or DBD mutant (C90A) substantially suppressed the luciferase activity, to further examine whether RORα interacts with NF-κB protein and transrepresses NF-κB-mediated transcription, we performed a reporter assay using NF-κB RE luciferase with the expression of RORα ΔAF2 mutant. The activation function 2 (AF2) domain of RORα is significant for activating direct downstream target genes which contain ROR binding element (RORE). The overexpression of RORα ΔAF2 mutant revealed that RORα indirectly transrepresses NF-κB RE via p50 (Figure 2C). Lipopolysaccharide (LPS) is one of the few known inducers of both the noncanonical and canonical pathways of NF-κB signaling activation. Silencing endogenous RORα by specific shRNAs caused further increase of NF-κB signaling downstream under LPS stimulation (Figure 2D). LPS treatment decreased mRNA levels of RORα by itself, further supporting the hypothesis that the LPS signal functions as an inducer of the NF-κB downstream pathway by downregulating RORα concomitantly (Figure S1A). LPS treatment had no significant effect in Jurkat cells, including increase of mRNA levels of *interferon γ* (*IFN-γ*). We used LPS stimulation as a dominant inducer of NF-κB signaling (Figure S1B). Therefore, we propose that the RORα-mediated attenuation of the NF-κB target gene expression in a DNA-binding-independent manner is fundamental to T cell activation.

To validate whether RORα is critical for the attenuation of NF-κB signaling to decrease cholesterol levels in T cells, we performed a chromatin immunoprecipitation (ChIP) assay with anti-RORα and HDAC3 antibodies in cells. The data obtained from the RORα-mediated transrepression of NF-κB allowed us to further investigate whether the loss of RORα was responsible for the hyperactivation of NF-κB target genes by using primary mouse embryonic fibroblasts (MEFs) prepared from RORα deficient staggerer (sg) mice, which had a spontaneous loss-of-function mutation in the RORα gene. Previous studies showed that promoters of *Acat1* and *Abcg1* have NF-κB binding sites from promoter analysis [39,40,41]. ChIP assay revealed that the recruitment of RORα and HDAC3 on the NF-κB target promoters was remarkably increased in control cells, but not in *RORα* KO (*sg*) or *p50* KO cells (Figure 2E), indicating that RORα-mediated transcriptional repression of NF-κB target genes is critical for the cholesterol metabolism. These data suggest that the RORα-mediated attenuation of NF-κB target gene expression, such as *Acat1* and *Abcg1*, is important for the reactivation of T cells.

### 2.3. Stimulating RORα Activities Enhances the Effector Responses of CD8^+^ T Cells through Cholesterol Esterification

**Figure 3:**
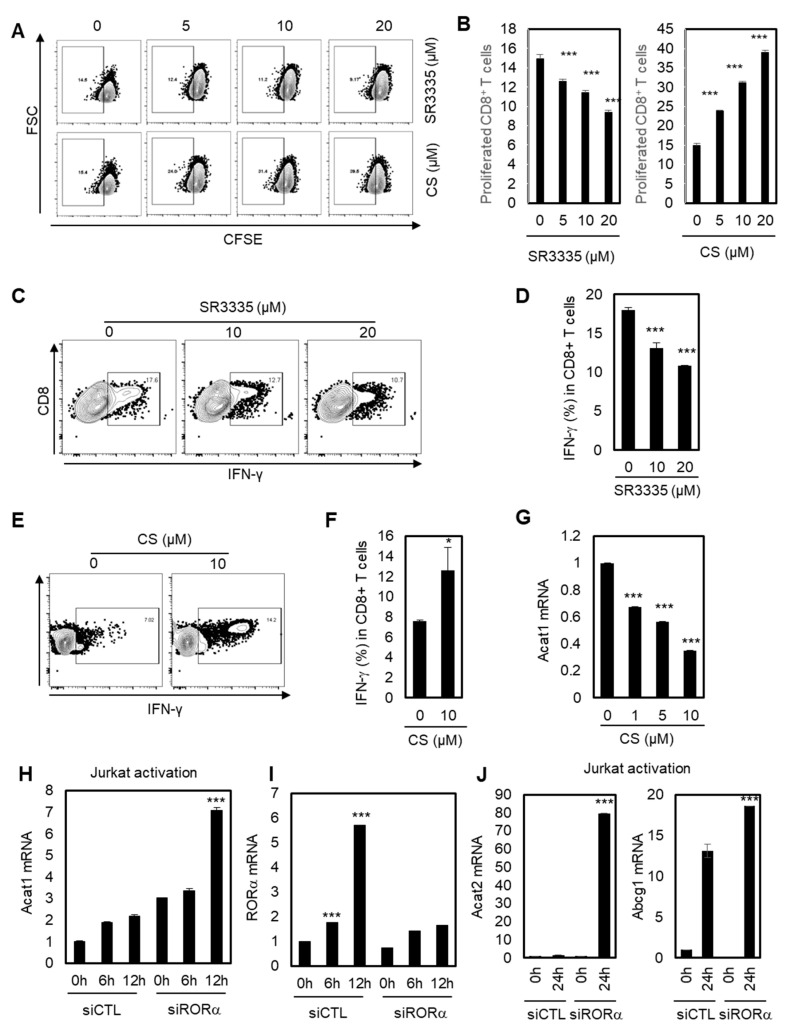
RORα potentiates CD8^+^ T cells through cholesterol esterification. (**A**) Dot plot analysis for the proliferation of CD8+ T cells treated with CS at doses of 5, 10, and 20 μM, or SR3335 at doses of 5, 10, and 20 μM. (**B**) Quantification of dot plat analysis in Figure 3A. The *p*-value was calculated by a *t*-test (*** *p* < 0.001). (**C,E**) IFN-γ production of CD8^+^ cells treated with 0, 10, and 20 μM of SR3335 (C) or 0 and 10 μM of CS (E). (**D,F**) Quantification of IFN-γ production of CD8^+^ cells treated with SR3335 (D) in Figure 3C or CS (F) in Figure 3E. The *p*-value was calculated by a *t*-test (* *p* < 0.05, *** *p* < 0.001). (**G**) Transcriptional level of *Acat1* in CD8^+^ cells treated with CS. The *p*-value was calculated by a *t*-test (*** *p* < 0.001). (**H**–**J**) Jurkat cells were transfected with either siRNA for control (siCTL) or siRNA of RORα, and the cells were activated at the indicated times. Transcriptional levels of *Acat1* (H), RORα (I), and Acat2/Abcg1 (J) were measured. The *p*-value was calculated by a *t*-test (*** *p* < 0.001).

A recent study showed that inhibiting cholesterol esterification potentiates the effector function of CD8^+^ T cells [42,43]. We hypothesized that RORα may regulate CD8^+^ T cells based on data showing that RORα acts as a corepressor for cholesterol esterification via NF-κB element-dependent promoter regulation such as *Acat1* and *Abcg1*. To validate this hypothesis, we first tested whether the proliferation and IFN-γ production of CD8^+^ T cells were affected by RORα activities. As shown in Figure 3A,B, inhibiting RORα activity using SR3335 reduced the proliferation of CD8^+^ T cells, while the potent agonist for RORα, CS, significantly enhanced it. In addition, SR3335 decreased the expression of IFN-γ in CD8^+^ T cells in a dose-dependent manner (Figure 3C,D). By contrast, CS significantly increased the expression of IFN-γ in CD8^+^ T cells (Figure 3E,F), supporting the hypothesis that enhanced RORα activity can increase the effector responses of CD8^+^ T cells. Next, we demonstrated that improved RORα activation using CS reduced mRNA levels of *Acat1*, indicating that RORα enhances the effector responses of CD8^+^ T cells by decreasing cholesterol esterification (Figure 3G). We also confirmed that *Acat1* mRNA expression was upregulated upon T cell activation, and that this upregulation was further enhanced by RORα knock-down, compared to control siRNA (siCTL) transfected T cells, showing the function of RORα to be a repressor for cholesterol esterification (Figure 3H). Consistent with the data in Figure 1A, *RORα* mRNA levels were significantly upregulated for 6 and 12 hrs upon activation of T cells; however, RORα targeting siRNA treatment was not able to enhance the transcript levels of *RORα* with T cell activation (Figure 3I). Additionally, knockdown of RORα also increased the activated mRNA levels of *Acat2* and *Abca1*, as expected (Figure 3J). Furthermore, SR3335 treatment specifically increased mRNA levels of *PD-1* in a dose-dependent manner in Jurkat cells, supporting the anticancer effect of T cells by reducing the activity of RORα (Figure S2). Taken together, it was shown that RORα regulates the effector responses of CD8^+^ T cells by inhibiting cholesterol esterification in a NF-κB-dependent manner, as evidenced by increased proliferation and IFN-γ production, with reduced *Acat1* mRNA expression, upon RORα activation.

### 2.4. Elevated Cholesterol Levels in the Plasma Membrane by Increased RORα Activation May Modulate the Function of CD8+ T Cells

**Figure 4:**
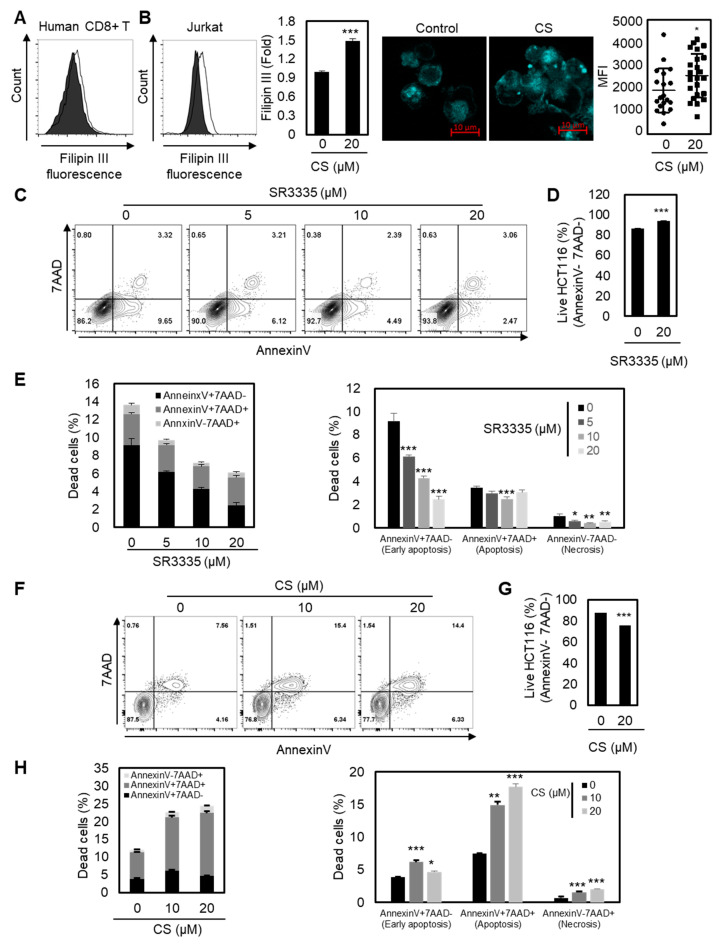
Enhanced cholesterol level by RORα may potentiate the antitumor activity of CD8^+^ T cells. (**A**,**B**) Cholesterol quantification of CD8^+^ T cells (**A**) or Jurkat (**B**) cells by Filipin III staining. CD8^+^ T cells or Jurkat cells were activated in the presence of CS 20 μM. The *p*-value was calculated by a *t*-test (*** *p* < 0.001). (**C**,**D**, and **E**) Quantification of cell death in colon cancer. HCT116 cells were cocultured with CD8^+^ T cells pretreated with SR3335 for three days. The *p*-value was calculated by a *t*-test (* *p* < 0.05, ** *p* < 0.01, *** *p* < 0.001). (**F**–**H**) Quantification of cell death in cancer. HCT116 cells were cocultured with CD8^+^ T cells pretreated with CS for three days. The *p*-value was calculated by a *t*-test (* *p* < 0.05, ** *p* < 0.01, *** *p* < 0.001).

ACAT1-deficient CD8^+^ T cells showed enhanced cholesterol levels in the plasma membrane and stronger T cell receptor signaling for T cell activation and proliferation [28]. Therefore, we next sought to determine the underlying mechanism of the enhanced effector function of CD8^+^ T cells with RORα activation by measuring the increased cholesterol contents in the plasma membrane of T cells. As shown in Figure 4A,B, the plasma membrane cholesterol level of T cells was substantially enhanced upon RORα activation, indicating a higher chance of stronger TCR signaling. These data suggest that RORα-mediated transcriptional repression of cholesterol esterification is critical for cholesterol levels in the plasma membrane in CD8^+^ T cells.

Finally, we assessed whether the reprogrammed cholesterol metabolism of CD8^+^ T cells by either RORα inhibition or activation controls cancer progression in vitro. Based on the data shown, we hypothesized that RORα inhibition attenuates the effector responses of CD8^+^ T cells, thereby reducing antitumor efficacy. Furthermore, we believed that RORα activation may enhance the activation of CD8^+^ T cells, leading to the inhibition of cancer progression. To test this hypothesis, we cocultured colorectal cancer cells HCT116 with CD8^+^ T cells pretreated with either SR3335 or CS, and measured apoptosis by FACS analysis. As expected, the apoptosis of cancer cells was reduced when it was cocultured with CD8^+^ T cells treated with RORα inhibitor SR3335 (Figure 4C–E). However, CD8^+^ T cells with RORα activation by CS enhanced the effector responses of CD8^+^ T cells, thereby inhibiting the viability of cancer (Figure 4F–H). Taken together, RORα-mediated regulation of cholesterol metabolism may contribute to the activation of T cell cytotoxicity.

## 3. Discussion

In recent years, there has been significant interest in the clinical application of cytotoxic T cells due to their central role in antitumor immunity in the tumor microenvironment [25,44,45]. In comparison to targeting cancer only, targeting both cancer and the cancer microenvironment has been demonstrated to offer benefits in cancer treatment. With a growing interest in immunotherapy for cancer treatment, the study of the reactivation of the cytotoxicity of CD8^+^ T cells has become one of the most attractive research areas in the development of anticancer therapeutics [46,47]. As potential therapeutic strategies, several immune checkpoint inhibitors have been developed for cancer treatment. In colorectal cancers, anti-PD-1 antibody therapy improves median overall survival in mismatch-repair-deficient patients, even though the response rates are not very high [48]. Also, blocking T cell immunoreceptor with Ig and ITIM domains (TIGIT) has emerged as a potential strategy to boost anticancer immunity [49]. Although various inhibitors targeting immune checkpoints to promote anticancer immunity have shown clinical benefits, we still need to improve the response rates of the treatment.

Targeting T cell metabolism can provide complementary benefits in current immune therapies with distinct mechanisms. Specifically, the function of traditional lipid metabolism drugs has been investigated to test the regulatory function of cholesterol in CD8^+^ T cell activation. Yang, et al. (2016) showed that the inhibition of cholesterol esterification by the Acat1 inhibitor avasimibe could enhance the effector function of CD8^+^ T cells [25]. It was also shown that lovastatin, an inhibitor of cholesterol biosynthesis, inhibits CD8^+^ T cell proliferation [50]. Additionally, atorvastatin, an HMG-CoA reductase inhibitor, was demonstrated to affect CD8^+^ T cell activation [51]. However, the effect of lipid metabolic reprogramming on T cell activation is controversial. It has been shown that cholesterol negatively regulates both the effector function of Tc9 cells and CD8^+^ T exhaustion in the tumor microenvironment [41]. Even though the detailed underlying mechanisms of modulating lipid metabolism associated with the effector function of CD8^+^ T cells need to be further investigated, targeting T cell metabolism could be an attractive target in cancer treatments.

Currently, there are no clinical trials related to RORα for cancer treatment, even though it has been shown to be a tumor suppressor in many preclinical studies [52,53]. However, increasing evidence demonstrates that RORα may also be a potential target in other diseases. RORα has been shown to be a key regulator for controlling hepatic lipid homeostasis [42]. RORα specifically binds to PPARγ target promoter with HDAC3 for transcriptional regression of PPARγ [43]. It was also demonstrated that RORα controls the inflammatory signaling network, which is implicated in inflammatory bowel disease (IBD) [3]. The function of RORα was also shown in the regulation of T cells. RORα promotes Th17 differentiation and regulates allergic skin inflammation via regulatory T cells [18,54]. Therefore, therapeutic strategies modulating RORα activity may be beneficial for the treatment of various human diseases. RORα has been shown to be a key regulator for controlling hepatic lipid homeostasis [55]; it specifically binds to the PPARγ target promoter with HDAC3 for transcriptional regression of PPARγ [56]. Therefore, it has been proposed that therapeutic strategies modulating RORα activity may be beneficial for the treatment of metabolic disorders.

**Figure 5:**
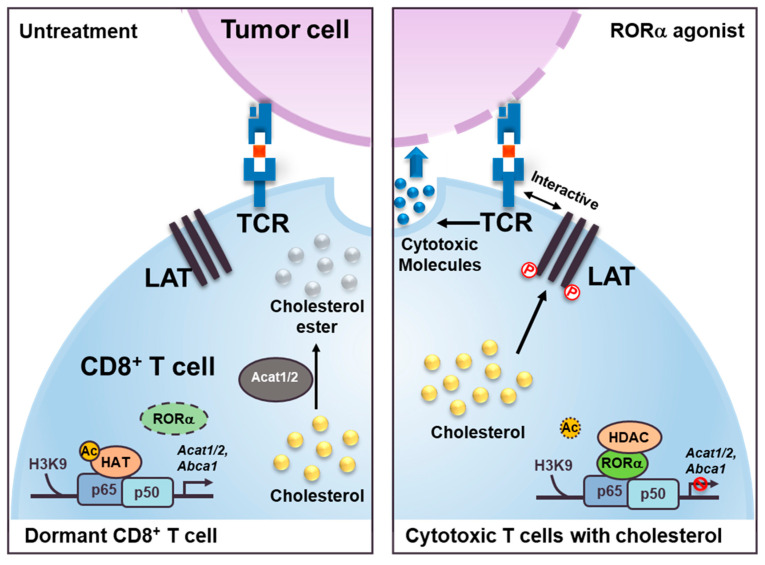
Schematic models show RORα agonist effect as inducing cholesterol metabolism in cytotoxic T cells. The models illustrate the role of RORα in CD8^+^ cytotoxic T cells. RORα agonist attenuates NF-κB signaling via HDAC recruitment on the *Acat1/2* and *Abca1* promoters for changing the status of T cells from dormant (left) to cytotoxic (right). These increased levels of cholesterol in the cell membrane of cytotoxic T cells induce a clustering of TCRs that interact with the antigens presented on the membrane of the cancer cells. Increased levels of cholesterol in cytotoxic T cells also trigger phosphorylation (P) of the linker of activated T cells (LAT), which, with TCRs, functions to stimulate the formation of immunological synapses.

In this study, we propose the modulation of RORα activity as a new therapeutic strategy for cancer immunotherapy. Numerous NRs are involved in cholesterol, including LXRα. We have shown that RORα is one of the dominant NRs connecting cholesterol metabolism and immunotherapy. We first identify RORα as a positive regulator of CD8^+^ T cells. The activation of RORα with CS developed an unusually stimulated effector response of CD8^+^ T cells. The necrosis and apoptosis of cocultured colon cancer cells with T cells were significantly increased upon RORα agonist treatment. RORα acts as a checkpoint, specifically inhibiting NF-κB-mediated transcriptional activity in the cholesterol metabolism signaling pathway, which is essential for the activation of T cells (Figure 5). Previously, our group found a correlation between RORα and HDAC3 in lipid homeostasis and genome-wide global promoter cooccupancy of RORα and NF-κB in intestinal epithelial cells [3,57]. However, connecting HDAC-RORα-NF-κB in activated T cells in the tumor microenvironment was the first approached; this connection is critical for crosstalk between cholesterol metabolism and the cytotoxicity of T cells. Our study provides a detailed mechanism, at the transcriptional level, of cholesterol esterification and the cholesterol efflux genes that are crucial for the control of sufficient cholesterol levels in T cells. The success or failure of CD8^+^ T cells surrounding cancer cells depends on the potentiation of cholesterol levels and the balance of cholesterol metabolism in T cells [28,58]. The upregulation of RORα appears to contribute to successful T cell activation by the attenuation of NF-κB activation in the cholesterol esterification and efflux pathways. Our results suggest distinct cholesterol-regulatory roles of RORα in the control of antitumor effects in CD8^+^ T cells. The modulating activity has been demonstrated to offer benefits in the treatment of cancer and metabolic disorders, and we now show that it can offer additional benefits in terms of enhancing anticancer immunity. RORα activation can be used to complement current immune therapies via a different mechanism.

## 4. Materials and Methods

### 4.1. Cell Culture

Human CD8^+^ T cells (HLA-A2-positive, STEMCELL, Vancouver, BC, Canada) were purchased. The cells were expanded by a 1:10 ratio of beads from the human T cell activation/Expansion kit (Miltenyi Biotec, Bergisch Gladbach, Germany). Every three days, the media, containing 20 units/ml of recombinant interleukin -2 (CORNING, Corning, NY, USA), was changed. CD8^+^ T cells will, hereafter, refer to human CD8^+^ T cells. Both CD8^+^ T cells and Jurkat cells were cultured with high glucose RPMI1640 (GIBCO, Waltham, MA, USA) containing 10% heat-inactivated fetal bovine serum, i.e., FBS (GIBCO), 1% Penicillin-Streptomycin; PS (GIBCO), L-glutamine, HEPES, and ß-mercaptoethanol. HCT116 (HLA-A2-positive), Jurkat cells, and NF-κB-luciferase reporter Jurkat cells were cultured with RPMI1640 media containing L-glutamine (GIBCO), 10% FBS, and 1% PS. NF-κB-luciferase reporter Jurkat cells were kindly provided by Dr. Park, Yoon from Korea Institute of Science and Technology. Jurkat cells (2~5 × 10^5^ cells/well) were activated using a T Cell Activation/Expansion Kit (Miltenyi Biotec); the ratio of cell-to-bead was 2:1.

### 4.2. Antibodies

The following commercially available antibodies were used: Anti-RORα (PA5-11224) was purchased from Thermo Fisher Scientific (Waltham, MA, USA), anti-HDAC3 (ab7030) was from Abcam (Cambridge, UK), anti-GFP (sc-9996) was from Santa Cruz biotechnology (Dallas, TX, USA), and anti-Flag antibody was from Sigma ST. Louis, MO, USA). Flow cytometric analysis for CD8^+^ T cells was performed with antihuman CD8a-PE (SK1, Biolegend, San Diego, CA, USA) and IFN-γ-APC (B27, Biolegend, San Diego, CA, USA). The analyze the apoptosis of cancer, HCT116 was stained by anneinxV-APC (BD Biosciences, San Jose, CA, USA) and 7AAD (BD Biosciences). All data were acquired by flow cytometry (CytoFlex, Beckman Coulter, Brea, CA, USA) and analyzed with FlowJo software (Tree Star, Ashland, OR, USA). Data are representative of three independent experiments.

### 4.3. The Measurement of Cholesterol Metabolic Regulation by RORα in T Cells

RORα and γ mRNA expression were measured at 6 and 12 hrs postactivation in Jurkat cells with antihuman CD3/28 antibodies (Miltenyi Biotec). Genes for cholesterol efflux (*Abcg1* and *Abca1*), cholesterol esterification (*Acat1* and *Acat2*), cholesterol synthesis (*DHCR24*, *HMGCR*, and *SREBP2*), and cytotoxic cytokine (*Granzyme B*) were measured in Jurkat cells treated with SR3335 (1, 5, 10, and 20 μM), SR1078 (1, 5, and 10 μM), or cholesterol sulfate; CS (1, 5, 10, and 20 μM). RORα knock-down was performed by the Neon™ transfection system with negative control siRNA (siCTL) or RORα siRNA (siRORα) with 50 nM. Validated siCTL and siRORα were purchased from Bioneer. AccuTarget™ Negative Control siRNA (Bioneer, Daejeon, Korea) was used as siCTL. siRORα sequences: 5′-CUCAGAACAACACCGUGUA-3′ and 3′-UACACGGUGUUGUUCUGAG-5’.

### 4.4. The Measurement of NF-κB Regulation by RORα

NF-κB-luciferase reporter Jurkat cells were treated with SR1078 (TOCRIS) or SR3335 (Caymanchem, Ann Arbor, MI, USA) for 1 or 2 days. Luciferase was reacted using a EZ™ Luciferase Assay System (enzynomics, Daejeon, Korea), according to the manufacturer’s instructions. Luminance was measured using a microplate reader (GloMax^®^ Discover Microplate Reader, Promega, Madison, WI, USA).

### 4.5. Reporter Assays

Using a luciferase system (Promega), the luciferase activity was measured using a luminometer 48 h after transfection, and normalized by β-galactosidase expression. Values are expressed as the means ± standard deviations of at least three independent experiments.

### 4.6. RORα Antagonist or Agonist Treatment on CD8^+^ T Cells

CD8^+^ T cells with antihuman CD3/28 antibodies (Miltenyi Biotec) were treated with 1, 5, 10, or 20 μM RORα antagonist (SR3335) or agonist (CS) for three days. HCT116 was cocultured with CD8^+^ T cells treated with RORα antagonist or agonist for three days. Intracellular staining protocol was performed according to the manufacturer’s instructions (BD Biosciences). Briefly, CD8^+^ T cells were treated with Golgi stop (BD Biosciences), phorbol 12-myristate 13-acetate (Invivogen, Invivogen, San Diego, CA, USA), and Ionomycin (Sigma-Aldrich) for 6 hr. A flow cytometric analysis of CD8^+^ T cells was performed with antihuman CD8a-PE (SK1, Biolegend) and IFN-γ-APC (B27, Biolegend), and granzyme B-FITC (GB11, BD Pharmingen) was performed according to the manufacturer’s instructions (BD Biosciences). Data acquired by flow cytometry (CytoFlex, Beckman Coulter) were analyzed with FlowJo software (Tree Star). Data are representative of three independent experiments. For proliferation measurements, CD8^+^ T cells were stained with CFSE kit (Invitrogen, Carlsbad, CA, USA), according to the manufacturer’s. Briefly, CD8^+^ T cells were cultured with 1 uM CFSE in PBS at 37 °C for 30 min. Then, the cells were cultured with complement media at 37 °C for 10 min. CFSE stained cells were activated with anti-CD3/28 beads for 2 d. Proliferation of CD8^+^ T cells was analyzed by flow cytometry.

### 4.7. Cell Proliferation Assay

CD8^+^ T cells were stained using a CellTrace^TM^ CFSE kit (invitrogen) according to the manufacturer’s instructions. Briefly, CFSE 1 uM were cultured with CD8^+^ T cells in PBS at 37 °C for 30 min. Then, the cells were cultured with complement media at 37 °C for 10 min. CFSE stained cells were activated with anti-CD3/28 beads for two days. The proliferation of CD8^+^ T cells was analyzed by flow cytometry.

### 4.8. Quantitative RT-PCR

Total RNAs were extracted using Trizol (Invitrogen), and reverse transcription was performed from 2.5 μg total RNAs using a M-MLV cDNA Synthesis kit (Enzynomics, Daejeon, Korea). The quatity of mRNA was determined using an ABI prism 7500 system or BioRad CFX384 with SYBR TOPreal qPCR 2x PreMix (Enzynomics). The quantity of mRNA was calculated using ddCt method, and Gapdh and β-actin were used as controls. All reactions were performed in triplicate. The following mouse primers were used in this study: β-actin; forward (fwd) 5′-TAGCCATCCAG GCTGTGCTG-3′, reverse (rev) 5′-CAGGATCTTCATGAGGTAGTC-3′; Gapdh; fwd 5′-CAT GGCC TTCCGTGTTCCTA-3′, rev 5′-CCTGCTTCACCACCTTCTTG A-3′; RORα; fwd 5′-CGGTGCGCAGACAGAGCTAT-3′, rev 5′-CCACAGATCTTGCATGGAATAATT-3′; RORγ; fwd 5′-TGGACCACCCCCTGCTGAGAAGG-3′, rev 5′-CTTCAATTTGTGTTCTCATGACT3′; Abcg1; fwd 5′-AAGGTGTCCTGCTACATCAT-3′, rev 5′-CAGTATCTCCTTGACCATTTC-3′; Acat1; fwd 5′-ATGCCAGTACACTGAATGATGG-3′, rev 5′-GATGCAGCATATACAGGAGCAA-3′; Abca1; fwd 5′-TGCAAGGCTACCAGTTACATT-3′, rev 5′-TTAGTGTTCTCAGGATTGGCT-3′; Acat2; fwd 5′-GCGGACCATCATAGGTTCCTT-3′, rev 5′-ACTGGCTTGTCTAACAGGATTCT-3′; DHCR24; fwd 5′-ACTCATTAGCTGTGTGACTC-3′, rev 5′-CAACAAGAGCTACCACTTAC-3′; HMGCR; fwd 5′-CTATGCTGGTCAGAAATAAC-3′, rev 5′-AGTAAGGAGGAGTTACCAAC-3′; SREBP2; fwd 5′-GACATCATCTGTCGGTGGTG-3′, rev 5′-GGGCTCTCTGTCACTTCCAG-3′.

### 4.9. ChIP Assays

The ChIP assay was conducted as previously described [9,10]. Quantitative PCR was used to measure the enrichment of bound DNA, and the value of enrichment was calculated relative to input and ratio to IgG. All reactions were performed in triplicate.

### 4.10. Constructions

The plasmid RORα DBD and ΔAF2 mutants were constructed as previously described [10].

### 4.11. Statistical Analysis

All experiments were performed independently at least three times. Values are expressed as mean ± SD. Significance was analyzed using a two-tailed, unpaired *t*-test. A *p*-value of less than 0.05 was considered statistically significant (* *p* < 0.05, ** *p* < 0.01, *** *p* < 0.001).

## 5. Conclusions

In summary, we investigated signaling crosstalk which is critical for the epigenetic control of the antitumor effect in the tumor microenvironment. The RORα-dependent attenuation of the NF-κB signaling pathway not only plays an important role in the proper regulation of target genes in human primary T cells, but also exerts cytotoxic effects on CD8^+^ T cells, eventually forming immunological synapses. We identified *Acat1*, *Acat2*, and *Abca1* as critical transcriptional target genes of the RORα/HDAC complex through their transrepressive activities on target genes. Furthermore, the treatment of natural and artificial agonists of RORα revealed that the epigenetic activation of RORα is a prerequisite for cholesterol homeostasis via the inhibition of NF-κB signaling in T cells. We provide new insights into the importance of epigenetic regulation and the dynamics of the antitumor effects of CD8^+^ T cells. To our knowledge, this is the first conclusive report to identify of a key nuclear receptor, i.e., RORα, in T cells that dynamically modulates cholesterol homeostasis in cytotoxic T cells through epigenetic regulation. As activation of RORα by an inhibitor has an important impact on the treatment of solid tumors via the regulation of cholesterol metabolism, the discovery of our epigenetic control of the tumor environment will greatly advance our understanding of therapeutic strategies in cancer treatment, including colon cancers.

## Supplementary Materials

The following are available online at https://www.mdpi.com/2072-6694/12/7/1733/s1, Figure S1: LPS treatment effects on mRNA levels of RORα and IFN-γ in each MEFs and Jurkat cells, Figure S2: SR3335 treatment increased the mRNA levels of PD-1. Transcriptional levels of *PD-1* in Jurkat cells treated with SR3335.

## Abbreviations

RORα:	Retinoic acid-related orphan receptor α
HDAC:	Histone deacetylase
PRRs:	Patterns recognition receptors
CD8:	Cluster of differentiation 8
TCR:	T cell receptor
CS:	Cholesterol sulfate
