# Supplementary material for: RORα Regulates Cholesterol Metabolism of CD8+ T Cells for Anticancer Immunity
Source: Cancers (Basel). 2020 Jun 29;12(7):1733. doi: 10.3390/cancers12071733 (PMC7407186; doi:10.3390/cancers12071733)
Supplement: Supplementary file 1 [file cancers-12-01733-s001.pdf]

RORα Regulates Cholesterol Metabolism of CD8+ T Cell for Anti-Cancer Immunity

In Kyu Lee, Hyerin Song, Hyerim Kim, Ik Soo Kim, Na Ly Tran, Sang-Heon Kim, Seung Ja Oh and Ji Min Lee

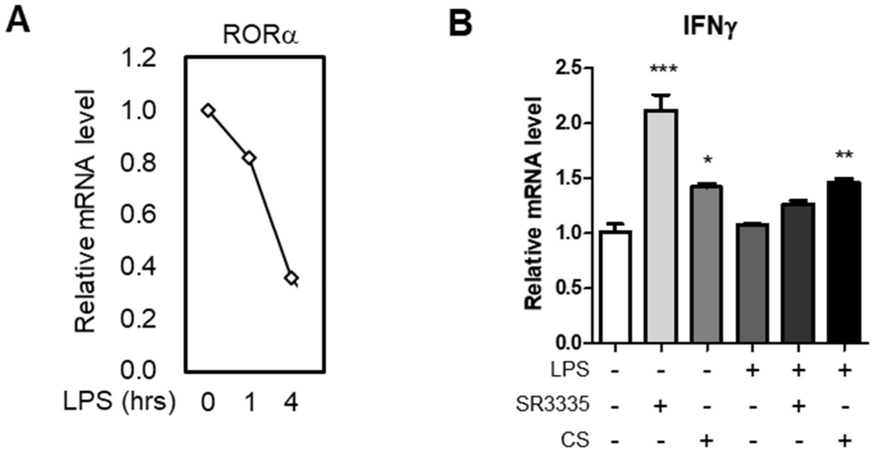

Figure S1. LPS treatment effects on mRNA levels of RORα and IFN-γ in each MEFs and Jurkat cells. (**A**) Transcriptional levels of mouse *RORα* in LPS-mediated stimulated MEFs as indicated activation times. (**B**) Transcriptional levels of IFN-γ in Jurkat cells treated with LPS, SR3335, and CS.

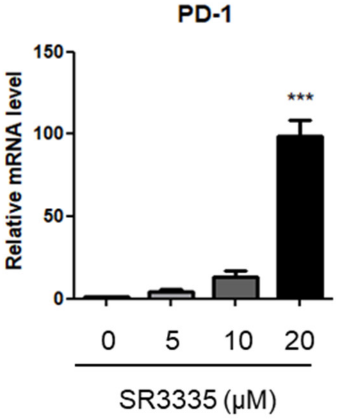

Figure S2. SR3335 treatment increased the mRNA levels of PD-1. Transcriptional levels of *PD-1* in Jurkat cells treated with SR3335.

© 2020 by the authors. Licensee MDPI, Basel, Switzerland. This article is an open access article distributed under the terms and conditions of the Creative Commons Attribution (CC BY) license (http://creativecommons.org/licenses/by/4.0/).
